# Diversification and spatial structuring in the mutualism between *Ficus septica* and its pollinating wasps in insular South East Asia

**DOI:** 10.1186/s12862-017-1034-8

**Published:** 2017-08-29

**Authors:** Lillian Jennifer Rodriguez, Anthony Bain, Lien-Siang Chou, Lucie Conchou, Astrid Cruaud, Regielene Gonzales, Martine Hossaert-McKey, Jean-Yves Rasplus, Hsy-Yu Tzeng, Finn Kjellberg

**Affiliations:** 1 Institute of Biology, University of the Philippines, Diliman, Quezon City, Philippines; 2 CEFE UMR 5175, CNRS—Université de Montpellier—Université Paul-Valéry Montpellier—EPHE, Montpellier, France; 3 INRA, UMR 1062 CBGP, Montferrier-sur-Lez, France; 4 Institute of Ecology and Evolutionary Biology, National Taiwan University, Taipei, Taiwan; 5 Institut d’Ecologie et des Sciences de l’Environnement de Paris—ECOSENS, INRA-UPMC, Versailles, France; 6 Department of Forestry and Natural Resources, National Chung-Hsing University, Taichung, Taiwan

**Keywords:** Biogeography, *Ceratosolen*, Mutualism, Philippines, Speciation

## Abstract

**Background:**

Interspecific interactions have long been assumed to play an important role in diversification. Mutualistic interactions, such as nursery pollination mutualisms, have been proposed as good candidates for diversification through co-speciation because of their intricate nature. However, little is known about how speciation and diversification proceeds in emblematic nursery pollination systems such as figs and fig wasps. Here, we analyse diversification in connection with spatial structuring in the obligate mutualistic association between *Ficus septica* and its pollinating wasps throughout the Philippines and Taiwan.

**Results:**

*Ceratosolen* wasps pollinating *F. septica* are structured into a set of three vicariant black coloured species, and a fourth yellow coloured species whose distribution overlaps with those of the black species. However, two black pollinator species were found to co-occur on Lanyu island. Microsatellite data on *F. septica* indicates the presence of three gene pools that broadly mirrors the distribution of the three black clades. Moreover, receptive fig odours, the specific message used by pollinating wasps to locate their host tree, varied among locations.

**Conclusions:**

*F. septica* and its black pollinator clades exhibited similar geographic structuring. This could be due originally to geographic barriers leading to isolation, local adaptation, and finally co-structuring. Nevertheless, the co-occurrence of two black pollinator species on Lanyu island suggests that the parapatric distribution of the black clades is now maintained by the inability of migrating individuals of black pollinators to establish populations outside their range. On the other hand, the distribution of the yellow clade strongly suggests an initial case of character displacement followed by subsequent range extension: in our study system, phenotypic or microevolutionary plasticity has allowed the yellow clade to colonise hosts presenting distinct odours. Hence, while variation in receptive fig odours allows specificity in the interaction, this variation does not necessarily lead to coevolutionary plant-insect diversification. Globally, our results evidence evolutionary plasticity in the fig-fig wasp mutualism. This is the first documentation of the presence of two distinct processes in pollinating fig wasp diversification on a host species: the formation of vicariant species and the co-occurrence of other species over large parts of their ranges probably made possible by character displacement.

**Electronic supplementary material:**

The online version of this article (doi:10.1186/s12862-017-1034-8) contains supplementary material, which is available to authorized users.

## Background

Interspecific interactions have long been assumed to play an important role in diversification. Competitive coevolutionary diversification among related species seems to be common while mutualistic coevolutionary diversification could be more exceptional [1]. Emblematic examples of allopatric speciation followed by competitive character displacement in secondary contact zones include *Anoles* lizards in Caribbean islands [2], Darwin’s finches [3] and sticklebacks [4]. Nevertheless, despite numerous putative cases, there are only few fully demonstrated examples of competitive character displacement [5].

Plants and their pollinators were proposed as models that should be prone to mutualistic coevolutionary diversification, because pollinators disperse the gametes of the plants whose flowers they visit [6]. Phylogenetic studies actually suggest that reciprocally-associated plant and pollinator clades often undergo asynchronous diversification consistent with a model of plants diversifying in response to sensory biases of their pollinators (e.g., [7]). Further, microevolutionary studies usually fail to test whether pollinators are evolving in response to the plants they visit. In nursery pollination systems, species-level specificity is often high, with most plant species usually locally pollinated by only one or a few host-specific insect species. This suggests that these mutualisms may coevolve. However, little is known about how speciation and diversification proceeds in emblematic nursery pollination systems such as figs and fig wasps (but see [8]) or in Phyllanthaceae and their pollinating moths [9]. Because of the intricate nature of these interactions, they have often been proposed as good candidates for diversification through co-speciation. However, comparing *Ficus* and their pollinating wasp phylogenies has shown that only 2/3 of the nodes are coevolved, and this is the most extreme example documented today of plant-insect codiversification [10]. At least in some of these systems, pollinator host shifts could be a mechanism that promotes diversification [1, 11]. In the *Yucca*-*Tegeticul*a interaction, results suggest that geographic isolation plays a predominant role in speciation for both *Yucca* and *Tegeticula*, with coevolution between them acting primarily to facilitate codivergence after one partner begins to diverge for other reasons [12, 13]. Similarly in the fig-fig wasp interaction, geographic isolation may also play an important role resulting in co-structuring, but does not necessarily result in congruent evolutionary histories of plant and insect [14].

While island systems are ideal situations to observe geographic isolation effects, they are also well represented in demonstrations of coevolutionary diversification as they provide repetitions of similar events thus providing statistical power [5]. Moreover, they have limited species diversity allowing related species to diversify into an assortment of ecological niches [5]. Because of the difficulty of re-assembly, it has been generally assumed that establishment of obligate mutualisms in islands is highly unlikely [15]. Nonetheless, existence of specialized mutualisms in island complexes defies this assumption. Indeed, the *Glochidion* tree-*Epicephala* moth mutualism is present throughout the tropical Pacific Islands [15]. Another example is the colonization of New Caledonia by section *Oreosycea* fig trees and their *Dolichoris* pollinators [16]. Although few, these studies show that dispersal and re-assembly of obligate mutualisms in islands is possible and results in evolutionarily stable interactions. Such situations allow analysing diversification patterns, some driven by obstacles to gene flow and others by host shifts [15, 17].

The intricate island systems of South East Asia and Australasia provide a diversity of situations in which *Ficus* species have diversified, and also in which some *Ficus* species have spread out over numerous islands [18]. Nevertheless, little is known about within-*Ficus* species diversification among islands and about associated pollinating wasp diversification. In insular systems, widespread *Ficus* species may be pollinated by different, more or less cryptic species of wasps in different parts of their range [17]. This is the case for *Ficus septica*, an insular fig species ranging from the Solomon Islands to the Ryukyu Islands and throughout insular South East Asia [17]. Previous work on *Ficus septica* suggested that coevolutionary diversification through competition could be at work among its pollinators, the *Ceratosolen bisulcatus* species complex [19]. Further, published data suggests the presence of among-island variation in receptive fig odour in *Ficus septica*, i.e. variation in the main message used by the wasps to locate their hosts (compare [19, 20]).

Our goal in this study was to describe the general pattern of diversification within the *Ficus septica*-pollinating wasps system and to demonstrate that it is consistent with the hypotheses (1) that geographic differentiation of receptive fig odours within *Ficus septica*, though present, is not a major biological barrier constraining the distribution of pollinator clades, (2) that coevolutionary diversification through competition followed by expansion through lineage sorting could be involved in an expansion of the *Ceratosolen jucundus* clade resulting in local coexistence of several pollinator lineages and (3) that the distribution of variation in plant and insects does not correspond to a generalized pattern of diversification through plant-insect reciprocal adaptation as geographic obstacles play a major role.

## Methods

### Study system

*Ficus septica* Burm.f. is a small free-standing tree distributed from the Solomon Islands and Northern Australia, throughout insular South East Asia, to Taiwan and the Ryukyu Islands. It belongs to subgenus *Sycomorus*, section *Sycocarpus* and it is functionally dioecious (functionally male trees bear figs that produce pollinating wasp offspring and pollen but no seeds, while female trees bear figs that produce seeds but neither wasp offspring nor pollen) [18]. Two polymorphic species (formally considered as subspecies) pollinate *F. septica*, namely *Ceratosolen bisulcatus bisulcatus* (Mayr) and *C. b. jucundus* Grandi [21]. Recent molecular analysis has demonstrated that these species are species complexes of closely related species [17], two of which were shown to co-occur in South Taiwan and in Luzon, Philippines, even at the fig-level [19, 22].

### General overview of sampling strategy

To get a broad image of the biogeographic distribution of *F. septica* pollinating wasp clades, we collected, sorted to morpho-species and sequenced pollinating wasps from all over the Philippines, from Taiwan and from Okinawa, and we examined published molecular data from the same region (Table 1). Genetic structuring within *F. septica* was investigated on a more limited scale, with four sampling points in the Philippines (one location in Mindanao, one in Negros, two in Luzon), one in Lanyu Island (60 km east of South Taiwan), and two in Taiwan (Table 1). The aim was to 1) confirm plant genetic homogeneity within islands and to detect potential genetic differentiation among islands and 2) to compare spatial genetic structuring within *F. septica* with the distribution of the different pollinator clades. Composition of the odours released by receptive figs (i.e. figs ready to be visited by pollinators), was investigated in four locations, one in Mindanao, one in Negros, one in Luzon and one in Taiwan, so as to represent the zones investigated for plant genetic structuring, and for which we also had information on pollinating wasps (Table 1). The aim was to detect whether differentiation in receptive fig odours, a highly selected trait of the plants, correlated or not with genetic differentiation in neutral markers of the plants. A complementary aim was to detect whether a given wasp clade could be associated with plant populations exhibiting divergent receptive fig odours, i.e. to detect whether geographic variation in receptive fig odours constrained the distribution of the different wasp clades.

**Table 1:** Collection sites for pollinator, leaf and fig odour samples

Country	Locality	*F. septica* pollinators	*F. septica* leaves	*F. septica* receptive fig odour
Japan	Okinawa	[55]		
Japan	Okinawa	This study		
Taiwan	North	This study, [17, 23]	This study	This study
Taiwan	Throughout the country	[56]		
Taiwan	South	This study, [17, 22]	This study	
Taiwan	Lanyu Island	[17, 22, 56]	This study	
Philippines	Northern Luzon	This study		
Philippines	Central Luzon	This study	This study	This study
Philippines	Southern Luzon	This study, [57]	This study	
Philippines	Panay Island	This study		
Philippines	Negros Island	This study	This study	This study
Philippines	Palawan Island	This study		
Philippines	Camiguin Island	This study		
Philippines	Mindanao Island	This study	This study	This study

Table 1 Legend: Sites are arranged in a north-to-south orientation. Also indicated are the references of the GenBank sequences (Additional file 8) that were blasted on our sequences for clade assignation. Pollinator genes analysed - [17, 50–52]: *COI*; [48]: *COI, 28S*; [49]: *18S, 28S, COI, cytb, Wg*; this study: *COI, cyt b, EF1α*. The *COI* sequences allow the assignment of every sequenced individual to clade and subclade recognised in this study

### Structuring into clades, distribution and phylogeny of *Ficus septica* pollinators

**Fig. 1:**
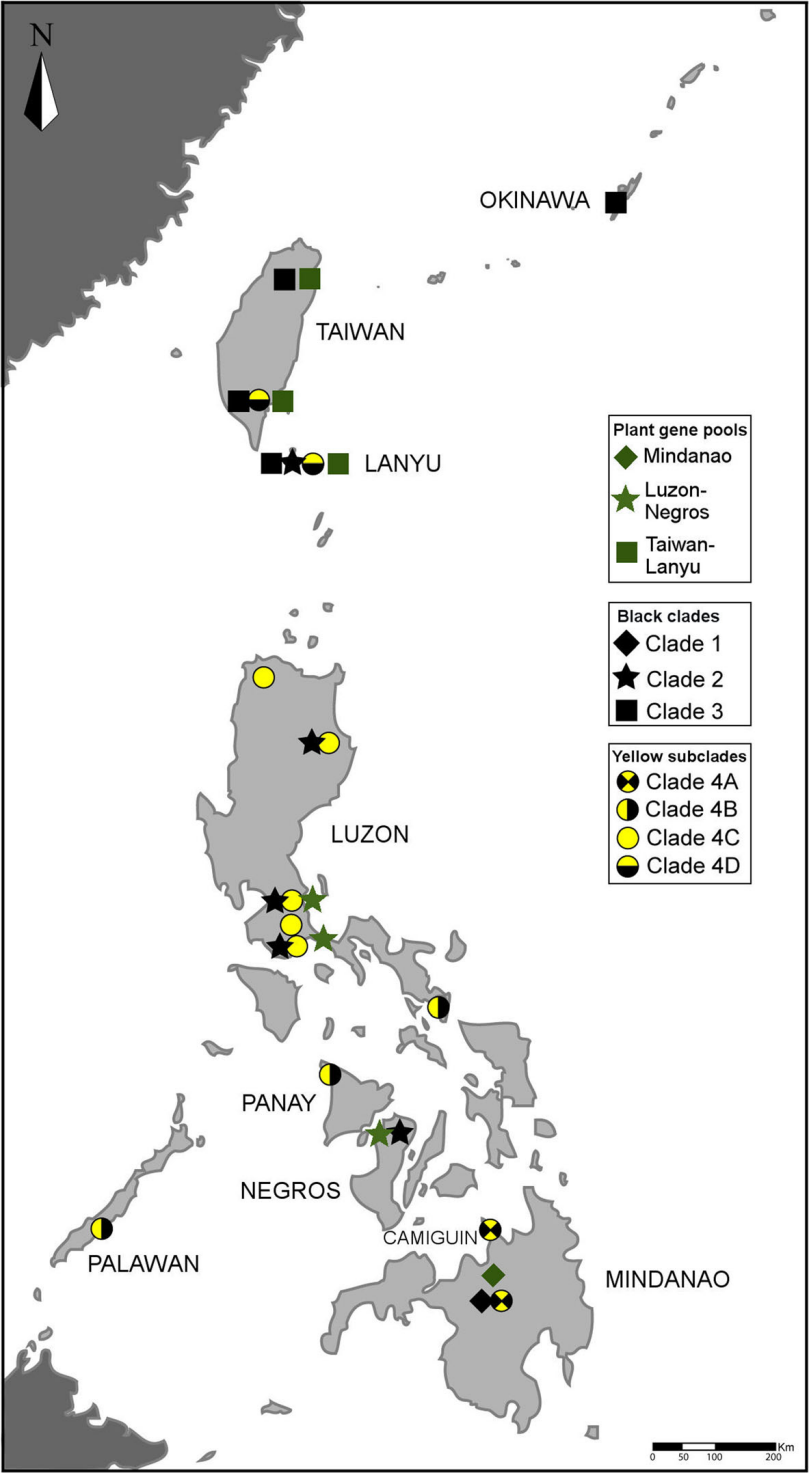
Geographic distribution of the *Ficus septica* gene pools and the different clades (and subclades) of the pollinators of *F. septica*

We sampled pollinator wasps from a large series of sites (Table 1, Fig. 1) from March 2011 to May 2014. Nearly ripe male figs were bagged and wasps were collected from the bags after their emergence from the figs. The samples were stored in vials with 70% ethanol. For each location, wasps were sorted to morpho-species and two to five wasp individuals of each morpho-species were sequenced for cytochrome *c* oxidase subunit I *(COI),* cytochrome *b (cyt b)* and elongation factor 1α (*EF1α*) (following [23, 24]). Extractions and amplifications were carried out at CBGP-INRA, Montferrier-sur-Lez, France.

### *Ficus septica* microsatellite analysis

To investigate genetic differentiation of *F. septica* among sites, we sampled leaves from seven study sites between April 2010 and September 2011. One leaf was collected from 17 to 37 trees per site in the Philippines and Taiwan (Table 1). Two pairs of collection sites were located on the same islands: on Luzon, they were about 100 km apart and on Taiwan, they were about 350 km apart. The leaves were oven-dried for two to three days and preserved dry. DNA was extracted from the leaf samples, amplified and genotyped (following [25]). Fourteen nuclear microsatellite markers specifically designed for the genus *Ficus* were used [25]. They were selected because of their high amplification rates and polymorphism (Additional file 1). Genotyping was carried out using an ABI 3730 Sequencer (Applied Biosystems).

### *Ficus septica* receptive fig odour collection and analysis

To collect odours from receptive figs, 5–20 *F. septica* trees were sampled per study site from March to September 2011. Scent extraction was carried out from 10.00 to 14.00 when scent emission is maximum [19]. As much as possible, male and female trees were equally represented in the sampling. Moreover, in the Philippines, fig scent collection was performed twice per site, at different seasons. Odours were collected using the headspace technique. Thirty to seventy receptive figs from each of the selected trees were placed inside a polyethylene terephthalate bag. The bag was closed for 30 min to allow the scent to accumulate. After the accumulation period, air was pumped out of the bag through two tubes, each with a chromatoprobe filter, for 5 min at a rate of 158 mL min^−1^. The volatile compounds were adsorbed into these filters, which were previously injected with known amounts of standard compounds (nonane and dodecane) to allow quantification of the volatiles. Empty bags were used as controls. The filters were placed in vials and stored at −18 °C until further analysis.

To identify and quantify the compounds present in the scent samples, filters were injected in a gas chromatograph (CP-3800, Varian Inc., Palo Alto, CA) coupled with a mass spectrometer (Saturn 2000, Varian; for identification of compounds) and equipped with a flame ion detector (for quantification of compounds). Samples were injected using a 1079 programmed temperature injector with a chromatoprobe thermodesorption kit (Varian). Temperature program and other conditions followed [19]. Component identification was based on the gas chromatograph (GC) retention indices of the compounds compared to those reported in literature [26] and through computer matching of mass spectra (MS) with NIST’98 MS library. These analyses were performed at the *Plate-Forme d’Analyses Chimique en Ecologie* of the LabEx CeMEB.

### Data analyses

For the phylogenetic analysis of pollinator wasp sequences, gene sequences were cleaned and edited in Geneious v5.5.9 and checked for presence of pseudogenes in Mega v6.06. We ran maximum likelihood (ML) analyses on the mitochondrial DNA dataset (*COI* - 1527 bp + *cyt b* - 734 bp) and on the nuclear DNA dataset (*EF1a* - 517 bp) using a GTR + Γ4 model with 1000 bootstrap replicates. We had 54 *COI*, 65 *cyt b* and 58 *EF1α* in-group sequences. We also used sequences of pollinator wasps of *F. botryocarpa, F. malayana* and *F. uncinata* as outgroups. In total, we had 13 *COI*, 10 *cyt b* and 8 *EF1α* outgroup sequences.

To quantify genetic variation between the *F. septica* populations, heterozygosity, mean number of alleles, and F_ST_ values were computed using genalex v6.5 [27] and spagedi v1.4 [28]. Exact tests of inter-population genetic differentiation were carried out using Fisher’s method in genepop v4.2 [29]. Finally, the microsatellite data was subjected to Bayesian clustering analysis using structure v2.3.4 [30] in order to determine the different genetic groups. The parameter set was a burn-in period of 100,000 steps and a run of 1,000,000 steps with no admixture, repeated five times. The number of individual clusters was determined using the Evanno method implemented in structure harvester [31]. We used the program STRUCTURE PLOT to visualize the Bayesian clusters [32].

Significant differences in total quantity of volatiles were tested using One-way ANOVA. For the subsequent analyses, relative abundance data was used. Fig scent composition was analyzed by constructing several models of MANOVA using geography, sex and season (or combinations of these) as explanatory variables. Significance values of each response variable were evaluated using the false discovery rate [33]. Spread of the variation in the data was visualized in a principal components analysis (PCA) performed using the package FactoMineR v1.31.5. A dissimilarity matrix using the Bray-Curtis index was also generated. Inter-population comparison of the blends was done by subjecting the Bray-Curtis indices to permutational analysis of variance (permanova) tests (permutations = 100,000) in the package vegan v2.3–3. All analyses were performed in R v3.2.1.

## Results

### *Ficus septica* microsatellite analysis

Genetic parameters averaged over 14 *Ficus septica* microsatellite loci used in this study are shown in Additional files 1 and 2. Expected heterozygosity (H_e_) and number of alleles per locus (Na) exhibited minimal variation in the seven sites (0.313–0.405) while private alleles (PA) varied more between islands. Exact tests of genotypic differentiation showed that all the population pairs, except the two from Luzon Island, were differentiated.

**Fig. 2:**
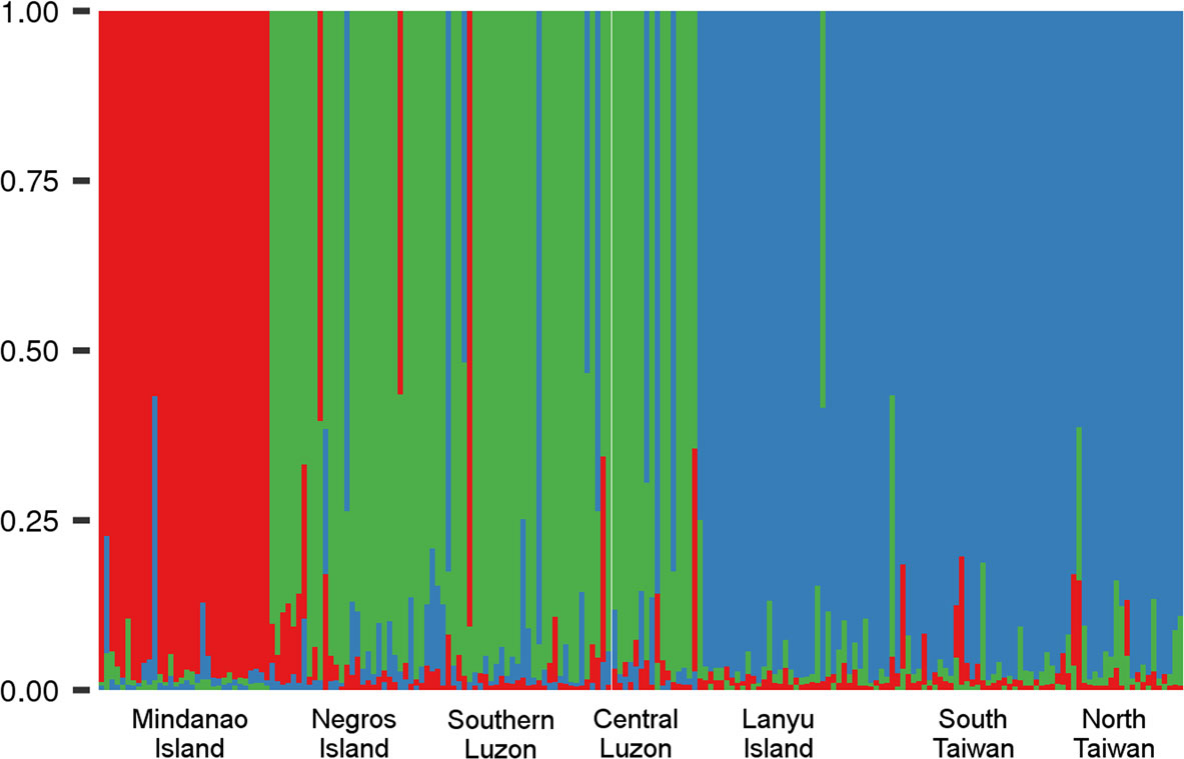
Plant genetics. Bar plots of the Bayesian clustering runs at the best K (3). Each column displays the probability of each individual to belong to each of the clusters. The three groups corresponded to i) Taiwan including Lanyu Island, ii) Luzon plus Negros Island, iii) Mindanao Island

F_ST_ values showed that the least differentiated populations were those from Luzon and those from Taiwan and Lanyu (Additional file 3). An intermediate homogeneity between the Luzon populations and the Negros population was evidenced by intermediate F_ST_ values demonstrating some genetic differentiation. The Bayesian clustering gave an optimal number of clusters, K, equal to 3 (Fig. 2). From South to North, the clusters gathered 1) the lone Mindanao population, 2) the Negros and Luzon populations, 3) the Lanyu Island population and the two Taiwan populations (Fig. 2).

### *Ficus septica* receptive odours

A total of 39 compounds were found in the scent of *F. septica*, with a little less than half classified as sesquiterpenes (Additional file 4)*.* However, the two dominant compounds, (E)-β-ocimene and (Z)-3-hexen-1-ol, were not sesquiterpenes but an acyclic monoterpene and a simple aliphatic, respectively. The most abundant fig odour component was α-pinene in Mindanao samples, (Z)-3-hexen-1-ol in Negros samples, (E)-β-ocimene in Central Luzon samples, and linalool in North Taiwan samples. Total amount of volatiles emitted did not differ significantly between locations, seasons and sexes (One-way ANOVA, *F*_10,45_ = 1.853, *P* = 0.078). Of the 39 compounds, 18 had a mean amount of at least 1% of the blend (Additional file 4). To allow significance testing, only the abundances of these 18 compounds were used for subsequent analyses.

**Fig. 3:**
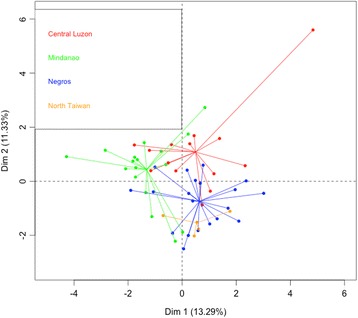
Plant odours. Principal Components Analysis of relative abundance of each compound in receptive fig odour showing groupings according to location. (North Taiwan – Yellow, Central Luzon – Red, Negros Island – Blue, Mindanao Island – Green). Odour profiles vary significantly between each of the four study sites (MANOVA, geography: approx. *F =* 5.45, *P* < 0.0001)

Using relative abundance values of the 18 compounds, we found that geography/location, season and their interaction factor were significant in explaining the variation in odour blend of receptive figs from Philippines (permanova, geography: *F* = 6.93, *P* < 0.0001; season: *F* = 2.55, *P* = 0.016; geography*season: *F* = 1.896, *P* = 0.026). Sex did not have a significant effect on scent variation (permanova, sex: *F* = 0.49, *P* = 0.868). When the Philippine dataset was partitioned into the three different sites, season had a significant effect in the scent variation in both Negros and Central Luzon, but not in Mindanao (permanova: Negros – *F* = 2.60, *P* = 0.012; Central Luzon – *F* = 2.89, *P* = 0.021; Mindanao – *F* = 1.20, *P* = 0.30; Additional file 5). In an analysis of all samples, including Taiwan, geography/location still showed a significant effect and sex still presented no effect on the blend (MANOVA, geography: approx. *F =* 5.45, *P* < 0.0001; sex: approx. *F* = 0.44, *P* = 0.967). Through pairwise comparisons of samples from different locations, it was found that the odour blend of the four *F. septica* populations significantly differed from each other (Additional file 6). This result was supported by the findings that the main compounds in the blends of the four sites differed (Additional file 7). PCA also reflected this differentiation (Fig. 3). The first two PCs explain about 24% of the variation in the data. The first axis separated the Mindanao samples from the other samples. The second axis separated the remaining three groups. In this representation, the barycenters of fig odours from Central Luzon and Negros were located next to each other.

In summary, data on receptive fig odour show that Mindanao, Negros, Central Luzon, and North Taiwan each have their own distinct odour profiles. These also show that geography/location is the main factor affecting fig scent variation, with the sex of the tree contributing very little, if at all, to the variation seen. Lastly, in the Philippines, season also accounted to some extent for scent variation in Negros and Central Luzon but not in Mindanao.

### Structuring into clades and phylogeny of *Ficus septica* pollinators

**Fig. 4:**
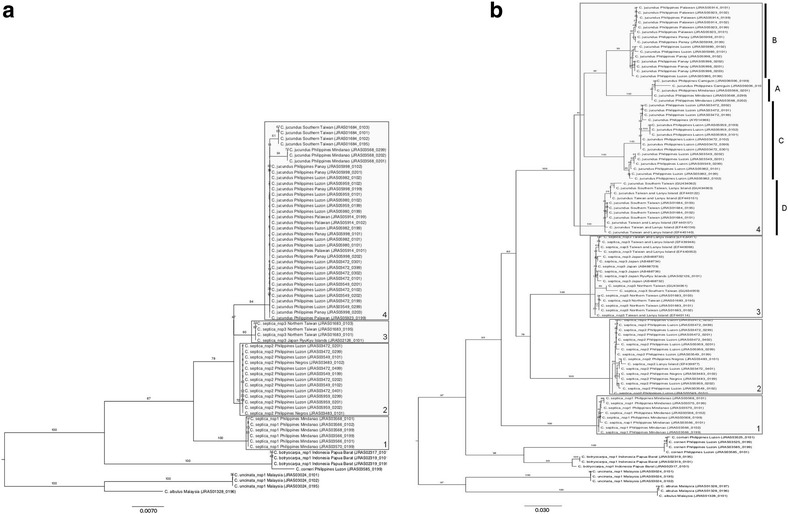
Insect phylogeny. Maximum likelihood (ML) trees from (**a**) nuclear DNA (*EF1α* gene sequences) and (**b**) mitochondrial DNA (*COI* and *cyt b* gene sequences) showing the four pollinator clades (clades 1 to 4) locally-associated with *Ficus septica*. Clade 4 in (**b**) is further subdivided into subclades 4A to 4D

Number of individuals sequenced, location of collection and GenBank accession numbers are given in Additional file 8. The phylogenetic trees derived from nuDNA (Fig. 4A) and mtDNA (Fig. 4B) show the presence of four major clades associated with *F. septica*. In our samples, three major pollinator clades (1–3) corresponded to black coloured morphs and the last major clade (clade 4) was composed of more or less yellow-coloured morphs, either dark backed-yellow sided (subclades 4A and 4B) or totally yellow (subclades 4C and 4D). Clades 1 to 3 followed a south to north distribution: clade 1 was present only in Mindanao, clade 2 ranged from Negros Island to Luzon (plus, according to *COI* sequences, one specimen collected on Lanyu Island and analyzed in [22]), and clade 3 occurred from Lanyu Island to Okinawa Island (Fig. 1). On the other hand, clade 4 showed less geographic structuring and was present throughout the Philippines up to South Taiwan (Fig. 1). Subclade 4A occurred in Mindanao and neighboring Camiguin Island, subclade 4B stretched from Palawan to the southern tip of Luzon. Subclade 4C ranged from Central to North Luzon and subclade 4D was present in Lanyu Island and South Taiwan. Clade 1 was found in sympatry with subclade 4A. Clade 2 coexisted with subclade 4C (Luzon) and with subclade 4D and clade 3 (Lanyu Island). Lastly, subclade 4B was not found in sympatry with any other clade. All previously published sequences from our sampling region fitted into the picture presented here. Samples from Indonesia and New Guinea [17] belonged to different clades from those recovered in this study.

## Discussion

### Genetic and odour structuring in *Ficus septica*

*F. septica* was spatially structured into three separate gene pools (from South to North): 1) the Mindanao population, 2) the Negros and Luzon populations, 3) the Lanyu Island population and the two Taiwan populations (Fig. 2). However, this genetic structuring was not reflected by the receptive fig odours because Mindanao, Negros, Central Luzon, and North Taiwan each had their own distinct odour profiles (Fig. 3). Hence receptive fig odours seem to evolve differently from neutral markers, suggesting selection. Beyond the case of *F. septica*, geographic variation in receptive fig odours has also been demonstrated in two fig species, *F. hispida* and *F. racemosa*. At least in *F. racemosa*, receptive fig odours varied between plants belonging to different gene pools and pollinated by different wasp species, while receptive fig odours did not differ between plants growing 800 km apart but belonging to the same gene pool and pollinated by the same wasp species [14, 34]. This similarity in fig odours suggests stabilising selection at work. Further, in the exceptional situation where two *Ficus* species freely share pollinators, their receptive fig odours are not distinguishable, suggesting selection by the wasps for evolutionary convergence of receptive fig odours [35, 36]. While we do not provide a direct demonstration that the difference in receptive fig odour reported here is meaningful for pollinating wasp attraction, more rapid evolution than neutral markers among islands is strikingly different from the stability and convergence of receptive fig odours documented in other *Ficus* species. Therefore we hypothesise that the differentiation of receptive fig odours in our study system is meaningful in terms of pollinator attraction.

The importance of receptive fig odours in pollinating wasp attraction has been largely demonstrated [37–39]. In contrast, variation within fig species [34] and among closely related species has been much less investigated [35, 36]. We document here seasonal variation in receptive fig odours in Luzon and Negros, but not in Mindanao, a difference that may be explained by limited seasonality in Mindanao comparatively to the two other islands [40]. This fits the very limited available data showing seasonal variation in receptive fig odours, measured within or between sexes depending on the species’ flowering phenology [20, 41]. Nevertheless, in all documented cases, differences between seasons remain limited, more limited than differences among species (except for *Ficus* species sharing pollinators [35, 36]) or than differences among islands for *F. septica*.

### Spatial structuring in *Ficus septica* and its pollinator clades

In *F. septica*, we show that gene pools do not neatly co-structure with receptive fig odours, as one gene pool (Luzon-Negros) corresponded to two distinct scent profiles (Luzon and Negros). This difference provides us with a new perspective to analyse pollinator structuring. Will pollinator species/populations reflect the geographic structuring of *F. septica* gene pools? Or will pollinators co-structure with receptive odour profiles, which are the main wasp attracting agents?

The pollinators of *Ficus septica* formed a monophyletic entity, structured into four major genetic clades that can be recognized morphologically, and four subclades within clade 4 that could not be distinguished morphologically, to the exception of colouration differences. According to available data on genetic distances within and among fig pollinating wasp species, the four major clades should be considered as distinct species while the subclades should be conservatively interpreted as within species variation [42]. The type of *Ceratosolen jucundus* was sampled on Mount Makiling [43], where we collected subclade 4C. Within this framework, *Ceratosolen bisulcatus jucundus* corresponds to clade 4 and is upgraded to species rank *Ceratosolen jucundus* (*stat. nov.*), while clades 1 to 3 correspond to three undescribed species that all appear different from *C. bisulcatus* (*stat. nov.*).

The three black coloured clades were allopatric to parapatric, presenting a geographic South-North zonation (Fig. 1). The black clades presented a zonation similar to that of the *F. septica* gene pools (Fig. 2). While sampling was not strictly comparable preventing formal statistical comparison, the similarity in spatial structure of clade distributions was striking. We hypothesize that this co-structuring is due to the presence of geographic barriers between the populations leading to isolation and subsequent diversification. *F. septica* and the black pollinator clades might have colonized the islands along the same South-to-North direction. Upon establishment in the different islands, populations must have been geographically isolated, subsequently facilitating local adaptation and diversification, such that the co-structuring between the fig and the wasps is evident now. Co-structuring, in this case, is thus driven more by geographic (and accompanying climatic) differences rather than by strict plant-insect reciprocal adaptation. Indeed, according to the Köppen-Geiger climate classification, the climates of Luzon and Negros are similar while the climates of Mindanao and Taiwan are more different [40]. This climatic differentiation is in broad concordance with the distributions of pollinator clades 1 to 3 and *F. septica* gene pools.

How do these patterns relate to receptive odour structuring? If an olfaction barrier maintains the specificity of the fig-wasp interactions, then we would expect each pollinator clade to recognize a single receptive odour profile. Nevertheless, geographic variation in receptive fig odours in *F. septica* has not precluded the presence of the following three situations: (1) coexistence of clades 2 and 3 on Lanyu Island, (2) presence of clade 2 in Luzon and Negros despite differing fig scent, and if our scenario is correct, (3) geographic expansion of clade 4 throughout the Philippines and up to Taiwan, bridging the different receptive fig odours produced by *F. septica*. These suggest either that wasps can rapidly evolve detection of new odours or that the wasps can still recognize receptive figs because the odour differences are limited. Hence, while receptive fig odour is a cue wasps use to locate their host figs [39], its differentiation among fig populations or among related *Ficus* species may allow phenotypic or micro-evolutionary plasticity in wasp odour recognition rather than being a constraint that would lead to strict insect-plant coevolutionary diversification. We may conjecture, for instance, that response to receptive fig odours, and maybe even perception of odours in wasps of clade 2 may vary between wasps originating from Negros, from Luzon and from Lanyu Island. Future experimental studies are needed to establish possible differential attraction to the various receptive odour compounds.

We therefore demonstrate here that structuring in *F. septica* and their pollinator clades is driven mainly by geographic (and climatic) factors rather than a species-specific olfaction barrier.

### Pollinator clade distribution and diversification

It should be noted that clades 2 and 3 are both present in Lanyu Island, which could be considered as their contact zone. Although these two clades are distributed over large stretches of sea presenting only few small islands, they still retain within-clade genetic homogeneity. This suggests sufficient effective numbers of migrants and hence sufficient dispersal. How then is the co-structuring maintained? What prevents expansion of clade 2 into South Taiwan (a distance of about 65 km from Lanyu Island) when it is able to hop along a series of islands up to 100 km apart from Luzon to Lanyu? Our working hypothesis is that migrant pollinators are unable to establish viable populations outside their realized range. This might be because local pollinators are more adapted to local conditions than the migrants, leading to competitive exclusion of migrating species. This pattern was also exhibited by the non-pollinating fig wasps associated with *Ficus rubiginosa* in Australia, where parapatric species pairs had smaller ranges than expected probably due to competition [44]. Therefore, the parapatric distribution of the black clades might be maintained by the inability of migrating species to establish populations outside their range.

Within this context, the distribution and incipient diversification of clade 4 is of particular interest. Clade 4 is present throughout the Philippines and extends 60 km northwards into South Taiwan, bridging almost the whole set of climates. However, the distribution of subclades is structured geographically with a South-to-North zonation. Also, subclades 4C and 4D are characterized by completely yellow colour in all the specimens we collected and their distributions totally overlap with the distributions of black coloured clades. Yellow colour is exceptional in diurnal fig pollinator species [19]. Individuals of subclade 4C have been demonstrated to exhibit very short survival times outside figs comparatively to co-occurring individuals of the black coloured clade 2 [19]. The authors interpreted the yellow colour of the clade as part of an adaptive syndrome based on short survival but high competitiveness, a “live fast, die young” strategy. Hence, clade 4 seems to have evolved a modified life strategy, which has enabled colonization of areas where *F. septica* is also pollinated by a black coloured clade and the subsequent stable coexistence of the two wasp species. This suggests recent range expansion into regions inhabited by clades 1 to 3 and ongoing differentiation into distinct gene pools. Such a scenario would involve initial ecological character displacement in a region of co-occurrence followed by subsequent colonization of the regions inhabited by the other black clades. In this sense, the initial clade 4 colonization of habitats already occupied by a black clade would qualify as ecological character displacement sensu stricto, while subsequent range extension would be made possible by differences evolved in allopatry, in a process of species sorting [5].

Interestingly, our results show the widespread co-occurrence of yellow *C. jucundus* wasps with the black pollinator clades. This has been previously reported, but on a much smaller scale [19, 22, 45]. Co-existence of reproductively isolated yellow and black pollinator clades were also documented in *F. rubiginosa* collected from Townsville, Australia [42, 46]. Aside from these studies and a few more [47], co-occurrence of sympatric, actively pollinating species over large areas has been rarely documented and their population genetics barely characterized. Our study fills this gap and we have proposed mechanisms on how this co-existence was achieved.

### Biogeography and adaptation in relation to other fig-wasp systems

How do our results compare with what is known about biogeography and adaptation in the fig-pollinating wasp system? The situation in *F. racemosa* resembles the situation of *F. septica* and its black pollinator clades, but on a broader scale. Indeed, the pollinators are structured into a set of wide-ranging vicariant species and the plant is structured along the same geographic lines into different gene pools, the limit of which seem to correspond to geographic barriers limiting gene flow [14]. Similarly, results on *F. hirta* and its pollinator in South East China show overall strong gene flow over continental expanses in both plant and insect, with genetically divergent populations for both plant and insect on Hainan island [48]. The authors suggested that the geographic barrier in itself was not sufficient to explain the data, and that isolation by adaptation, facilitated by a decrease in dispersal across a geographic barrier, had to be invoked [48]. In the data presented here, the presence of clade 2 in Lanyu Island supports this vision, as it shows that fig pollinating wasp species can achieve continuity across almost 400 km of sea, hopping from island to island with some over 100 km apart: the 30 km separating Hainan island from the continent can hardly be considered by themselves a major obstacle for fig wasp dispersal. Moreover, data on *F. pumila*, a creeping fig, and its pollinators in Eastern China showed some genetic isolation by distance in the plant [49], and separation of the pollinators into two wide ranging vicariant species. South of Shanghai, in the Zhoushan archipelago, within a context of some geographic obstacles to dispersal, a localized third pollinator species is present in combination with one of the wide-ranging species [50]. The two co-occurring species differ in precocity of emergence from figs after over-wintering, suggesting differences in temperature thresholds. This ecological differentiation may facilitate the coexistence of competing species [50], while the parapatric distribution of the two wide-ranging species is probably best explained by competitive exclusion in a context of strong gene flow limiting local adaptation in widespread species [51]. Finally, *F. rubiginosa* provides the most complex pattern of distribution of diverse pollinator species documented to date, a feature that may result from the range of the host tree encompassing a combination of two climatic gradients, a progressive North-South gradient and a steep East-West one [42, 46, 52]. Hence, the biogeographic patterns observed in fig-pollinating wasp associations tend to support a global vision in which the pollinators of wide ranging fig species, but not necessarily the fig species, are often structured into broad, geographically vicariant, genetically homogeneous entities. New lineages of pollinating wasp species may colonize such systems following a local event of ecological character displacement. This can be followed by subsequent colonization of parts of the range of the fig occupied by other pollinators through a process of species sorting resulting in the regional coexistence of two pollinating wasps on the same host. We were able to demonstrate these general emergent patterns for pollinating fig wasp species diversification in a relatively simple fig-wasp system. This leads us to suggest that within *Ficus* species, in an insular setting, fig-fig wasp diversification and co-structuring may be frequent. These patterns do not need to result from coevolutionary diversification [1]. Indeed, in our fig-fig wasp system, geographic barriers seem to have been the main driving factor leading to plant-insect co-structuring, a feature that does not necessarily entail parallel cladogenesis [14]. Interestingly, the co-occurrence of clades 2 and 3 in Lanyu Island suggests that barriers that might arise from coevolutionary diversification can be overcome, limiting its role in the process of joint plant-insect diversification.

The Philippines constitute a model island archipelago to investigate evolutionary processes of diversification [53]. Much focus has been on limited dispersal species, for which routes of colonization can be reconstructed using phylogenies. The results presented here on *Ficus septica* allow a different perspective. What biogeographic structure do we get in strong dispersal model species and what does it tell us about evolutionary processes at work in lower dispersal species? Our study may shed light on results such as those on shrews [54] as it supports a role for geographic barriers, but probably also for ecological character displacement followed by species sorting in diversification processes. Our results also emphasize the importance of climatic variation among islands as a force structuring diversity and diversification.

## Conclusions

We have shown that *F. septica* and its black pollinator clades exhibited similar geographic structuring. We hypothesize that this could be due originally to strong geographic (and climatic) barriers leading to isolation, local adaptation, and finally plant-insect co-structuring. We also show the co-existence of pollinating species in an area, which probably had been brought about by initial character displacement and subsequent range expansion. This is the first documentation of the presence of two distinct processes in pollinating fig wasp diversification on a host species: the formation of vicariant species and the co-occurrence of other species over large parts of their ranges probably made possible by character displacement. Lastly, we provide evidence that receptive fig odours do not constrain the specificity of the fig-pollinator wasp interactions.

## Additional files

Additional file 1: Characteristics of 14 microsatellite loci used in the study across all *Ficus septica* individuals. H_o_ - observed heterozygosity, H_e_ - expected heterozygosity, Na - number of alleles per locus, NA - Not Applicable. (DOCX 61 kb)

Additional file 2: Genetic parameters from 14 microsatellite loci of *Ficus septica*. *n* – number of leaves sampled, H_e_ - expected heterozygosity, Na - mean number of alleles per locus, PA - mean number of private alleles. (DOCX 51 kb)

Additional file 3: Genetic differentiation in *Ficus septica* populations as shown from F_ST_ values computed from 14 microsatellite loci. All F_ST_ values (besides between the two populations from Luzon) are significant at *P* < 0.05 using the individual permutation tests. TW – Taiwan; PH – Philippines. (DOCX 62 kb)

Additional file 4: Volatile organic compounds emitted by *Ficus septica* from the Philippines and North Taiwan. Compounds with more than 1% mean relative abundance are shown in boldface. (DOCX 79 kb)

Additional file 5: Plot of the mean relative abundance of the 18 most abundant compounds found in *Ficus septica* odours between two seasons (dry and rainy) in two Philippine sites: Central Luzon and Negros Island. (PDF 9 kb)

Additional file 6: Permutational analysis of variance (permanova) comparisons of the fig odour profiles (relative abundance) of *Ficus septica* populations from North Taiwan and three Philippine sites (Central Luzon, Negros Island, Mindanao Island) (*-significant difference at *P* < 0.05). Significant *P*-values for all comparisons show that each of the 4 sites has its own distinct odour profile. (DOCX 56 kb)

Additional file 7: Plot of the mean relative abundance of the 18 most abundant compounds found in *Ficus septica* odours from North Taiwan and three sites in the Philippines (Central Luzon, Negros Island, Mindanao Island): the most abundant compound is different in each of the sites. (PDF 9 kb)

Additional file 8: Sampling table for *Ceratosolen bisulcatus* wasps indicating collection locality and GenBank accession number for each sequence used for the phylogenetic analysis. (DOCX 108 kb)

## Abbreviations

ANOVA: analysis of variance
*COI*: cytochrome *c* oxidase subunit I
*cyt b*: cytochrome *b*
*EF1α*: elongation factor 1α
GC: gas chromatograph
GTR: general time reversible
H_e_: expected heterozygosity
MANOVA: multivariate analysis of variance
ML: maximum likelihood
MS: mass spectra
mtDNA: mitochondrial DNA
Na: number of alleles per locus
nuDNA: nuclear DNA
PA: private alleles
PC: principal component
PCA: principal components analysis
Permanova: permutational analysis of variance
