# Supplementary material for: Diversification and spatial structuring in the mutualism between Ficus septica and its pollinating wasps in insular South East Asia
Source: BMC Evol Biol. 2017 Aug 29;17:207. doi: 10.1186/s12862-017-1034-8 (PMC5576367; doi:10.1186/s12862-017-1034-8)
Supplement: Supplementary file 1 — Characteristics of 14 microsatellite loci used in the study across all Ficus septica individuals. Ho - observed heterozygosity, He - expected heterozygosity, Na - number of alleles per locus, NA - Not Applicable. (DOCX 61 kb) [file 12862_2017_1034_MOESM1_ESM.docx]

**Additional file 1**

Characteristics of 14 microsatellite loci used in the study across all *Ficus septica* individuals. H_o_ - observed heterozygosity, H_e_ - expected heterozygosity, Na - number of alleles per locus, NA - Not Applicable.

___________________________________________________________________________

**Locus Na Min-Max allele size H_o_ H_e_ Amplification**

**____________________________________________________________________________**

Car11 10 163-186 0.688* 0.664 1.00

Car1 11 187-210 0.751 0.694 0.72

Car3 5 113-126 0.017* 0.051 0.99

Car10 6 105-205 0.213* 0.396 0.72

Car2 6 89-212 0.071 0.066 0.84

Car5 3 74-80 0.156 0.171 1.00

Car6 6 81-95 0.160* 0.319 0.91

Car8 10 88-119 0.558* 0.545 0.94

Car7 2 200-206 NA NA 0.99

Car4 3 123-132 0.011 0.011 1.00

Car9 6 93-109 0.508* 0.566 0.99

Micr3 7 134-192 0.174* 0.335 0.99

Micr2 11 89-114 0.646* 0.699 0.99

Sur1 12 181-215 0.667* 0.681 0.99

*Significant difference (*P* < 0.05) between expected and observed heterozygosities.
