# Supplementary material for: Diversification and spatial structuring in the mutualism between Ficus septica and its pollinating wasps in insular South East Asia
Source: BMC Evol Biol. 2017 Aug 29;17:207. doi: 10.1186/s12862-017-1034-8 (PMC5576367; doi:10.1186/s12862-017-1034-8)
Supplement: Supplementary file 2 — Genetic parameters from 14 microsatellite loci of Ficus septica. n – number of leaves sampled, He - expected heterozygosity, Na - mean number of alleles per locus, PA - mean number of private alleles. (DOCX 51 kb) [file 12862_2017_1034_MOESM2_ESM.docx]

**Additional file 3**

Genetic parameters from 14 microsatellite loci of *Ficus septica*. *n* – number of leaves sampled, H_e_ - expected heterozygosity, Na - mean number of alleles per locus, PA - mean number of private alleles.

| **Location** | ***n*** | **H_e_** | **Na** | **PA** |
| --- | --- | --- | --- | --- |
| Taiwan – North | 30 | 0.361 | 3.50 | 0.43 |
| Taiwan – South | 30 | 0356 | 4.00 | 0.43 |
| Taiwan – Lanyu Island | 30 | 0.313 | 3.21 | 0.21 |
| Philippines – Central Luzon | 37 | 0.389 | 3.78 | 0.29 |
| Philippines – Southern Luzon | 27 | 0.366 | 3.57 | 0.14 |
| Philippines – Negros Island | 17 | 0.405 | 3.64 | 0.14 |
| Philippines – Mindanao Island | 32 | 0.353 | 4.29 | 0.79 |
