# Supplementary material for: Diversification and spatial structuring in the mutualism between Ficus septica and its pollinating wasps in insular South East Asia
Source: BMC Evol Biol. 2017 Aug 29;17:207. doi: 10.1186/s12862-017-1034-8 (PMC5576367; doi:10.1186/s12862-017-1034-8)
Supplement: Supplementary file 3 — Genetic differentiation in Ficus septica populations as shown from FST values computed from 14 microsatellite loci. All FST values (besides between the two populations from Luzon) are significant at P < 0.05 using the individual permutation tests. TW – Taiwan; PH – Philippines. (DOCX 62 kb) [file 12862_2017_1034_MOESM3_ESM.docx]

**Additional file 4**

Genetic differentiation in *Ficus septica* populations as shown from F_ST_ values computed from 14 microsatellite loci. All F_ST_ values (besides between the two populations from Luzon) are significant at *P* <0.05 using the individual permutation tests. TW – Taiwan; PH – Philippines.

| Location | TW – North | TW – South | TW – Lanyu Island | PH – Central Luzon | PH – Southern Luzon | PH – Negros Island | PH – Mindanao Island |
| --- | --- | --- | --- | --- | --- | --- | --- |
| TW – North | - |  |  |  |  |  |  |
| TW – South | 0.026** | - |  |  |  |  |  |
| TW – Lanyu Island | 0.051*** | 0.026** | - |  |  |  |  |
| PH – Central Luzon | 0.114*** | 0.143*** | 0.143*** | - |  |  |  |
| PH – Southern Luzon | 0.113*** | 0.170*** | 0.168*** | 0.016 | - |  |  |
| PH – Negros Island | 0.135*** | 0.178*** | 0.168*** | 0.084*** | 0.083*** | - |  |
| PH – Mindanao  Island | 0.203*** | 0.179*** | 0.193*** | 0.209*** | 0.242** | 0.173*** | - |
