# Supplementary material for: Diversification and spatial structuring in the mutualism between Ficus septica and its pollinating wasps in insular South East Asia
Source: BMC Evol Biol. 2017 Aug 29;17:207. doi: 10.1186/s12862-017-1034-8 (PMC5576367; doi:10.1186/s12862-017-1034-8)
Supplement: Supplementary file 4 — Volatile organic compounds emitted by Ficus septica from the Philippines and North Taiwan. Compounds with more than 1% mean relative abundance are shown in boldface. (DOCX 79 kb) [file 12862_2017_1034_MOESM4_ESM.docx]

**Additional file 5**

Volatile organic compounds emitted by *Ficus septica* from the Philippines and North Taiwan. Compounds with more than 1% mean relative abundance are shown in boldface.

| **Type** | **Compound** | **Mean Relative Abundance** |
| --- | --- | --- |
| Simple aliphatics | **(Z)-3-hexen-1-ol** | 15.02% |
|  | **3-hexenyl acetate** | 3.55% |
|  | **(E)-4,8-dimethylnona-1,3,7-triene** | 3.84% |
|  | **dodecene 1** | 1.67% |
|  | **(Z)-4,8-dimethylnona-1,3,7-triene** | 1.10% |
| Acyclic monoterpenes | **(E)-β-ocimene** | 19.14% |
|  | **(Z)-β-ocimene** | 1.28% |
|  | **linalool** | 4.90% |
|  | cis-pyranoid-linalool-oxide | <1% |
| Cyclic monoterpenes | **α-pinene** | 8.96% |
|  | **sabinene** | 6.10% |
|  | **eucalyptol** | 2.17% |
| Sesquiterpenes | **α-copaene** | 9.22% |
|  | **(E)-caryophyllene** | 3.69% |
|  | **δ-cadinene** | 1.53% |
|  | **α-humulene** | 1.03% |
|  | alloaromadendrene | <1% |
|  | α-ylangene | <1% |
|  | bicyclogermacrene | <1% |
|  | β-elemene | <1% |
|  | α-cubebene | <1% |
|  | δ-elemene | <1% |
|  | longifolene | <1% |
|  | β-selinene | <1% |
|  | sesquiterpene 4 | <1% |
|  | sesquiterpene 6 | <1% |
|  | sesquiterpene 7 | <1% |
|  | isoledene | <1% |
|  | cis-α-bergamotene | <1% |
| Benzenoids | **phenylethyl alcohol** | 2.15% |
|  | **o-guaiacol** | 1.26% |
|  | benzyl alcohol | <1% |
| Lactones | **3-methyl-2(5H)-furanone** | 2.13% |
| Unclassified | isophorol | <1% |
|  | Unknown 6 | <1% |
|  | Unknown 52 | <1% |
|  | Unknown 57 | <1% |
|  | Unknown 95 | <1% |
|  | Unknown 96 | <1% |
