# Supplementary figures and images for: Diversification and spatial structuring in the mutualism between Ficus septica and its pollinating wasps in insular South East Asia
Source: BMC Evol Biol. 2017 Aug 29;17:207. doi: 10.1186/s12862-017-1034-8 (PMC5576367; doi:10.1186/s12862-017-1034-8)

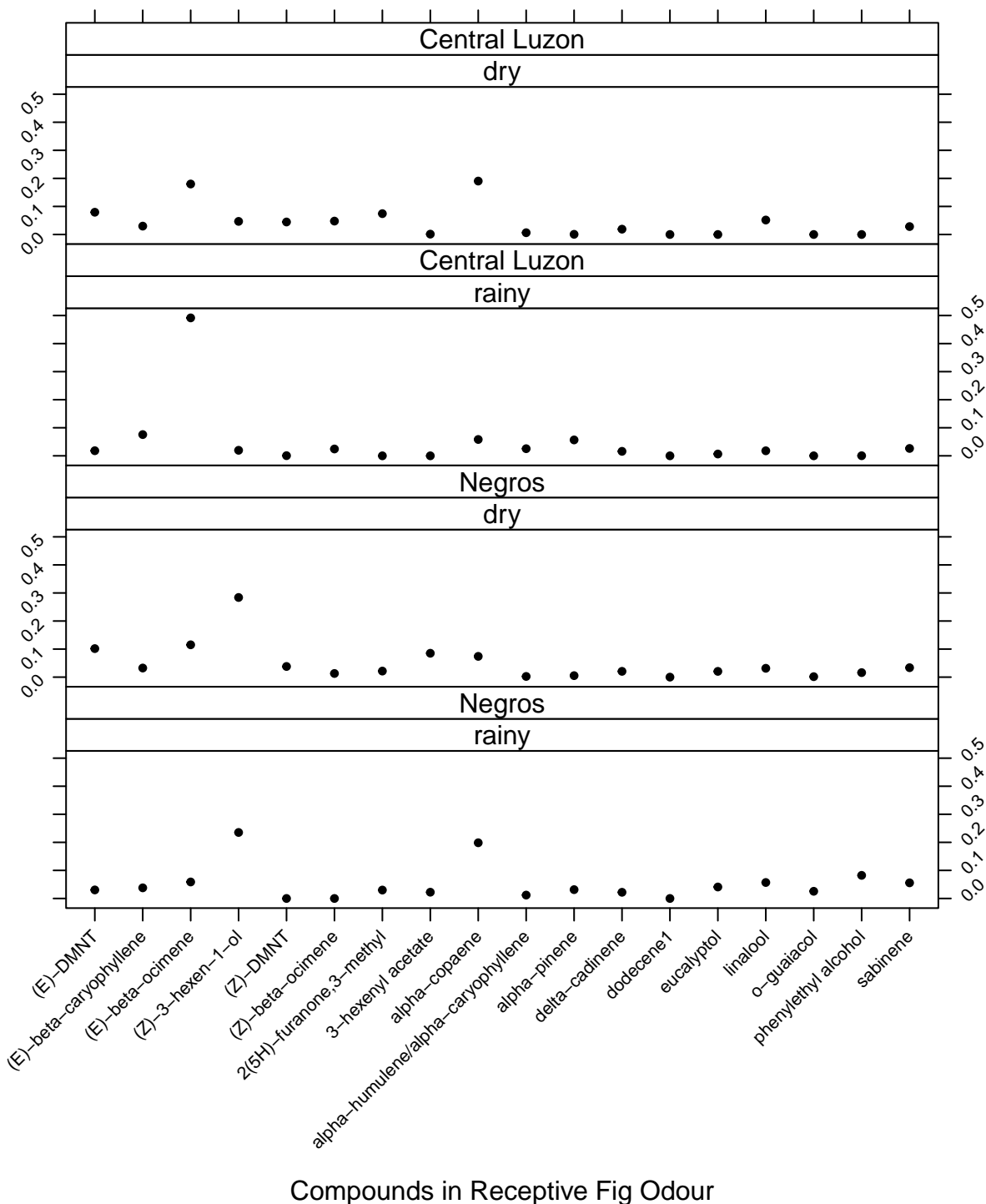

Supplement: Supplementary file 5 — Plot of the mean relative abundance of the 18 most abundant compounds found in Ficus septica odours between two seasons (dry and rainy) in two Philippine sites: Central Luzon and Negros Island. (PDF 9 kb) [file 12862_2017_1034_MOESM5_ESM.pdf]

Relative Amount

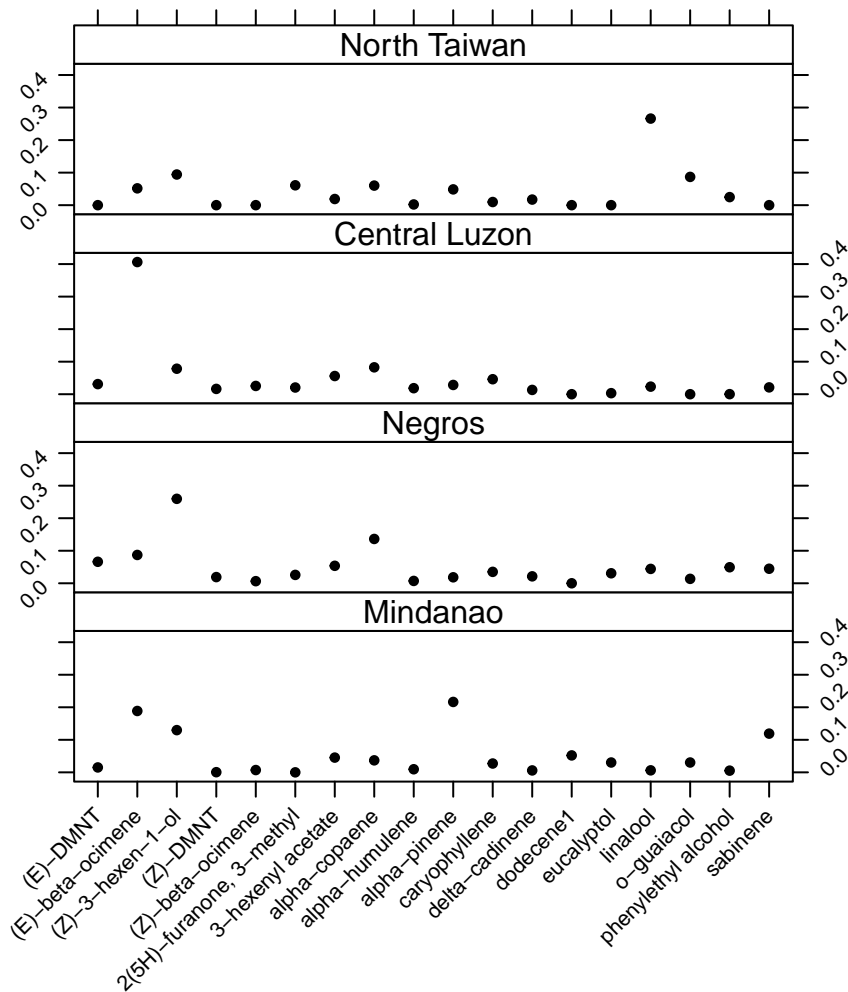

Compounds in Receptive Fig Odour

Supplement: Supplementary file 7 — Plot of the mean relative abundance of the 18 most abundant compounds found in Ficus septica odours from North Taiwan and three sites in the Philippines (Central Luzon, Negros Island, Mindanao Island): the most abundant compound is different in each of the sites. (PDF 9 kb) [file 12862_2017_1034_MOESM7_ESM.pdf]
