# Supplementary material for: Diversification and spatial structuring in the mutualism between Ficus septica and its pollinating wasps in insular South East Asia
Source: BMC Evol Biol. 2017 Aug 29;17:207. doi: 10.1186/s12862-017-1034-8 (PMC5576367; doi:10.1186/s12862-017-1034-8)
Supplement: Supplementary file 6 — Permutational analysis of variance (permanova) comparisons of the fig odour profiles (relative abundance) of Ficus septica populations from North Taiwan and three Philippine sites (Central Luzon, Negros Island, Mindanao Island) (*-significant difference at P < 0.05). Significant P-values for all comparisons show that each of the 4 sites has its own distinct odour profile. (DOCX 56 kb) [file 12862_2017_1034_MOESM6_ESM.docx]

**Additional file 7**

Permutational analysis of variance (permanova) comparisons of the fig odour profiles (relative abundance) of *Ficus septica* populations from North Taiwan and three Philippine sites (Central Luzon, Negros Island, Mindanao Island) (*-significant difference at *P*<0.05). Significant *P*-values for all comparisons show that each of the 4 sites has its own distinct odour profile.

| Populations Compared | *F* value | *P*-value |
| --- | --- | --- |
| North Taiwan – Central Luzon | *F*_1,17_=5.15 | 0.0003* |
| North Taiwan – Negros Island | *F*_1,23_=3.18 | 0.0025* |
| North Taiwan – Mindanao Island | *F*_1,20_=4.33 | 0.0005* |
| Central Luzon – Negros Island | *F*_1,32_=7.98 | <0.0001* |
| Central Luzon – Mindanao Island | *F*_1,29_=4.91 | <0.0001* |
| Negros Island – Mindanao Island | *F*_1,35_=6.49 | <0.0001* |
