# Supplementary material for: Diversification and spatial structuring in the mutualism between Ficus septica and its pollinating wasps in insular South East Asia
Source: BMC Evol Biol. 2017 Aug 29;17:207. doi: 10.1186/s12862-017-1034-8 (PMC5576367; doi:10.1186/s12862-017-1034-8)
Supplement: Supplementary file 8 — Sampling table for Ceratosolen bisulcatus wasps indicating collection locality and GenBank accession number for each sequence used for the phylogenetic analysis. (DOCX 108 kb) [file 12862_2017_1034_MOESM8_ESM.docx]

**Additional file 2**

Sampling table for *Ceratosolen bisulcatus* wasps indicating collection locality and GenBank accession number for each sequence used for the phylogenetic analysis.

| **Clade/ Subclade** | **Location** | **GenBank Accession Number** | | |
| --- | --- | --- | --- | --- |
|  |  | ***COI*** | ***cyt b*** | ***EF*** |
| Clade 1 | Mindanao, Philippines | KX668819, KX668820, KX668821, KX668822, KX668823, KX668824, KX668826, KX668827 | KX668754, KX668755, KX668756, KX668757, KX668758, KX668759, KX668762, KX668763 | KX668686, KX668687, KX668688, KX668689, KX668690, KX668694 |
| Clade 2 | Luzon, Philippines | KX668805, KX668806, KX668807, KX668808, KX668809, KX668813, KX668814, KX668815, KX668838, KX668839, KX668840, | KX668734, KX668735, KX668736, KX668740, KX668741, KX668742, KX668748, KX668750, KX668775, KX668776, KX668777 | KX668670, KX668671, KX668672, KX668676, KX668677, KX668680, KX668681, KX668703, KX668704, KX668705 |
| Clade 2 | Negros, Philippines | KX668810, KX668811, KX668812 | KX668743, KX668744, KX668745 | KX668678, KX668679 |
| Clade 2 | Lanyu Island | EF439977 |  |  |
| Clade 3 | Taiwan and Lanyu Island | EF439911, EF439948, EF440052, EF440068, EF440114, AY879563, KX668794, KX668795, KX668796 | KX668720, KX668721, KX668722 | KX668653, KX668654, KX668655 |
| Clade 3 | RyuKyu Islands, Japan | GU434061, KX668798 | KX668727 | KX668660 |
| Clade 4A | Mindanao, Philippines | KX668825 | KX668760, KX668761 | KX668691, KX668692, KX668693 |
| Clade 4A | Camiguin, Philippines |  | KX668790, KX668791 |  |
| Clade 4B | Palawan, Philippines | KX668829, KX668830, KX668831, KX668832, KX668833, KX668834 | KX668766, KX668767, KX668768, KX668769, KX668770, KX668771 | KX668696, KX668697, KX668698 |
| Clade 4B | Panay, Philippines | KX668847, KX668848, KX668849, KX668850, KX668851, KX668852 | KX668784, KX668785, KX668786, KX668787, KX668788, KX668789 | KX668712, KX668713, KX668714, KX668715, KX668716, KX668717 |
| Clade 4B | Luzon, Philippines | KX668841, KX668842, KX668843 | KX668778, KX668779, KX668780 |  |
| Clade 4C | Luzon, Philippines | AY014986, KX668803, KX668804, KX668816, KX668817, KX668818, KX668844, KX668845, KX668846 | KX668731, KX668732, KX668733, KX668737, KX668738, KX668739, KX668751, KX668752, KX668753, KX668772, KX668773, KX668774, KX668781, KX668782, KX668783 | KX668667, KX668668, KX668669, KX668673, KX668674, KX668675, KX668683, KX668684, KX668685, KX668700, KX668701, KX668702, KX668706, KX668707, KX668708, KX668709, KX668710, KX668711 |
| Clade 4D | Taiwan and Lanyu Island | EF440122, EF440136, EF440140, EF440151, EF440157, GU434062, GU434063 | KX668723, KX668724, KX668725, KX668726 | KX668656, KX668657, KX668658, KX668659 |
